# Knocking Down miR172f in the Hairy Roots of Grass Pea Increases β-ODAP Content and Induces Global Transcriptomic Reprogramming

**DOI:** 10.3390/genes17030311

**Published:** 2026-03-09

**Authors:** Xiaoning Liu, Xueping Zhang, Jianmeng Bai, Jiasheng Lv, Yingshan Jiang, Jiahui Zhan, Zhihong Yang, Rongze Han, Tingli You, Hao Ma, Ning Cao, Rongfang Lian, Shijun Wang, Yun Yue, Quanle Xu

**Affiliations:** 1 School of Medicine, Huanghe S & T University, Zhengzhou 450063, China; xiaoningliu2016@126.com; 2 College of Life Sciences, Northwest A&F University, Yangling 712100, China; 15104297730@163.com (X.Z.); baijianmeng@126.com (J.B.); 13909358252@163.com (J.L.); yingshanjiang@nwafu.edu.cn (Y.J.); zhan-jiahui@nwafu.edu.cn (J.Z.); y2359631211@163.com (Z.Y.); 15731355789@163.com (R.H.); youtingli2026@163.com (T.Y.); mahao806@163.com (H.M.); cndx121@163.com (N.C.); 3 Dingxi Academy of Agricultural Sciences, Dingxi 743000, China; gsdxlianrongfang@163.com; 4 Gansu Academy of Agri-Engineering and Technology, Lanzhou 730030, China; 18189566671@163.com

**Keywords:** β-N-oxalyl-L-α,β-diaminopropionic acid (β-ODAP), *Lathyrus sativus* L., BAHD3 acyltransferase, miR172f, transcriptomic analysis

## Abstract

**Background**: There is an abundance of the neuroactive β-N-oxalyl-L-α,β-diaminopropionic acid (β-ODAP) in grass pea (*Lathyrus sativus*), pea (*Pisum sativum*), and several Chinese traditional herbs such as *Panax notoginseng*. It is well known for its dose- and context-dependent effects on its toxicological characteristics (inducing neurodegenerative neurolathyrism upon excessive consumption) or for its pharmacological effects (including neuroprotection and wound healing). Therefore, reducing β-ODAP levels improves the safety profile of β-ODAP-containing species for utilization, whereas increasing them facilitates their isolation and purification. *Ls*BAHD3 acyltransferase, named after the first letter of BEAT benzylalcohol O-acetyltransferase (BEAT), anthocyanin O-hydroxycinnamoyltransferase (AHCT), anthranilate N-hydroxycinnamoyl/benzoyltransferase (HCBT), and deacetylvindoline 4-Oacetyltransferase (DAT), was proven to be β-ODAP synthetase. **Methods**: In this report, the interaction of miR172f with *LsBAHD3* was investigated through bioinformatic analysis and transient co-expression assays in *Nicotiana benthamiana*. Functions of miR172f in β-ODAP biosynthesis were also investigated through knockdown in the hairy roots of *L. sativus* and via transcriptomic analysis. **Results**: The results suggest that the knockdown of miR172f in hairy roots of *L. sativus* increased β-ODAP content via targets to *LsBAHD3*. In this process, protein ubiquitination, cysteine and methionine metabolism, enzyme regulator activity, and so on were associated with β-ODAP biosynthesis. **Conclusions**: These results identify miR172f as a novel regulator of β-ODAP biosynthesis through targeting of *LsBAHD3*, offering new insight into the gene expression of β-ODAP synthetase and the genetic network governing β-ODAP biosynthesis in *L. sativus*.

## 1. Introduction

Grass pea (*Lathyrus sativus* L.) is grown for human consumption or forage in many arid and semiarid regions for its general resistance to different biotic and abiotic stresses [1]. Nevertheless, incorporating *L. sativus* as a major component in the daily diet is a challenge because it accumulates the neuroactive toxin β-N-oxalyl-L-α,β-diaminopropionic acid (β-ODAP) [2]. β-ODAP is well known for its dual toxicological and pharmacological effects in a nutritional context or mode of application [3] and its abundance in pea (*Pisum sativum*) [3,4], *Panax notoginseng*, *P. ginseng*, *P. quinquefolium*, etc. [5,6,7]. The content of β-ODAP in *L. sativus* was affected by different biotic and abiotic stressors, such as drought, waterlogging, nutrient deficiencies, heavy metals, and salinity [8,9,10]. Therefore, β-ODAP biosynthesis needs to be regulated to utilize species containing β-ODAP.

The primary metabolism pathways, including alanine and nitrogen metabolism and cysteine and sulfur metabolism, are highly involved in β-ODAP metabolism [11,12,13]. Several genes encoding serine acetyltransferase (SAT), β-cyanoalanine synthase (β-CAS), BAHD3 acyltransferase (BAHD3), and acyl-activating enzyme 3 (AAE3) were identified in β-ODAP biosynthesis [3,12,14,15,16,17,18]. Of these, *Ls*BAHD3 was proven to be β-ODAP synthetase (BOS), which catalyzes L-DAP (2, 3-diaminopropionic acid) oxalylation using oxalyl-CoA as the donor to form β-ODAP [3,15,19].

MicroRNAs participate in modulating plant development, stress resistance, and bioactive compound production [20,21,22,23]. For instance, miR160, miR167, miR169, and miR171 are involved in pathways that respond to nitrogen stress [23]; miR395 family members are general components of sulfur metabolism and its regulatory network [24,25,26]. miR172 family members play essential roles in regulating plant growth, biosynthesis of several secondary metabolites like phenylalanine and phenolic acids, and mediating response to environmental stress, including drought, salinity, and heavy metal exposure [27,28,29]. As the content of β-ODAP is strongly correlated with nitrogen and sulfur metabolism, different biotic and abiotic stresses and microRNAs involved in β-ODAP biosynthesis should be investigated.

In this study, miR172f was identified as a novel regulator of β-ODAP biosynthesis through targeting of *LsBAHD3*. The results will provide new insight into the gene expression of β-ODAP synthetase and the genetic network governing β-ODAP biosynthesis in *L. sativus*.

## 2. Materials and Methods

### 2.1. Determination Overexpression of the LsBAHD3 Gene in the Hairy Roots of P. sativum via RT-PCR

The transgenic hairy root line of *P. sativum OE LsBAHD3*–*13* was produced by Zhang et al. [3]. Total RNA was extracted from the hairy roots of *OE LsBAHD3*–*13* using RNAiso plus (Takara, Dalian, China) according to the manufacturer’s protocol. Then, cDNAs of *OE LsBAHD3*–*13* were obtained by reverse transcription of the extracted total RNA using the FastKing RT Kit (with gDNase) (Tiangen, Beijing, China). PCR amplification was conducted via the 2× IProof polymerase mix under the following conditions: one cycle of 95 °C for 5 min; 30 cycles of 95 °C for 30 s, 52 °C for 30 s, and 72 °C for 40 s. The products were visualized via electrophoresis on 1% agarose gel. Primers used in this study are presented in Table 1.

### 2.2. Identification of miR172f and Detection of Its Expression Level via RT-qPCR

miRNAs targeting the *LsBAHD3* gene were identified through the online website psRNATarget (https://www.zhaolab.org/psRNATarget/ accessed on 15 December 2022). Then, the expression level of miR172f in *OE LsBAHD3*–*13* was detected via RT-qPCR, and U6 was used as an internal reference. The stem-loop primer was used for reverse transcription of miR172f (Table 1), and the ^ΔΔ^Ct method was employed to normalize and quantify the relative fold changes in three biological replicates.

### 2.3. Transient Expression of miR172f and LsBAHD3 in Nicotiana benthamiana

*LsBAHD3* or pre-miR172f was cloned into vectors pTF486-eGFP or pOT2-Poly-cis separately. *Agrobacterium tumefaciens* GV3101 carrying the recombinant pTF486-BAHD3-eGFP or pOT2-miR172f was infiltrated in pairs into 5-week-old leaves of *N. benthamiana* and cultured for 3 d in the dark. Microscopic analyses were conducted using a Leica DMi8 Inverted Microscope (Leica, Wetzler, Germany), and GFP fluorescence was captured using an excitation wavelength of 488 and an emission wavelength of 530 nm. *N. benthamiana* leaves infiltrated with *A. tumefaciens* GV3101 carrying pTF486-eGFP or POT2 were used as the control.

### 2.4. Knockdown of miR172f in L. sativus Hairy Roots

*A. rhizogenes* C58C1 competent cells were transformed with *pFGC5941-pOT2-STTM-miR172f* or *pOT2-poly-cis* separately and cultured on YEB solid medium containing 50 μg/L rifampicin and kanamycin at 28 °C to induce the hairy roots of *L. sativus* according to the method reported by Zhang et al. [3]. The positive transgenic hairy roots were identified by RT-qPCR using U6 as the internal reference.

### 2.5. Determination of β-ODAP Content via HPLC

The β-ODAP content in miR172f knocked-down hairy roots of *L. sativus* was detected via HPLC as described by Jiao et al. [30] using an Alliance™ HPLC System (Waters, Milford, MA, USA) equipped with a column of Symmetry C18 (4.6 × 250 mm, 5 μm). The mobile phase was acetonitrile and 0.1 M HAc-NaAc (17:83, V/V, pH 4.4), and the flow rate was set at 1.0 mL/min.

### 2.6. Transcriptomic Analysis

Transcriptomic analysis was performed on three independent biological replicates of the transgenic *L. sativus* hairy root line STTM-1 with miR172f knocked-down and the negative control at Lc-Bio Technologies (Hangzhou, China) Co., Ltd. The differentially expressed genes (DEG) between samples were identified according to statistically significant differences with the threshold of false-discovery-rate (FDR)-adjusted *p*-value < 0.05 and |log2Fold Change (FC)| ≥ 1. Principal component analysis (PCA) was performed with the R package gmodels, and the heatmap was plotted using the pheatmap package (v1.0.12) in R (v4.1.2). The R package Weighted Gene Co-Expression Network Analysis (WGCNA) was used to infer highly co-expressed gene modules from the DEGs. Gene set enrichment analysis (GSEA) was performed using the GSEA software (v4.1.0) with the absolute value of normalized enrichment score (NES) > 1 and *p* < 0.05.

### 2.7. Statistical Analysis

All values are expressed as the mean ± standard deviation from three individual experiments. Data were analyzed with IBM SPSS Statistics 27 software. For any one-way analysis of variance (ANOVA) test at the *p* < 0.01 or *p* < 0.05 levels, the asterisk indicates statistically significant differences between samples.

## 3. Results

### 3.1. Reduction in Expression Level of miR172f in the P. sativum Hairy Roots of OE LsBAHD3–13

**Figure 1:**
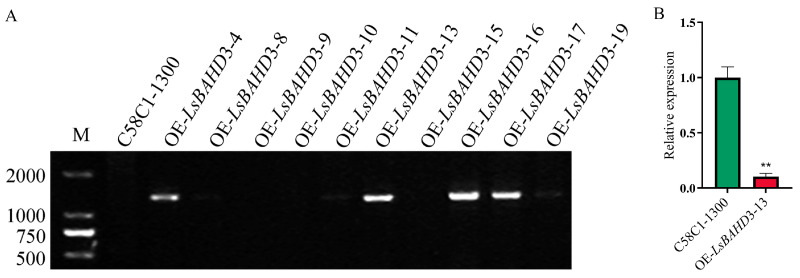
Identifying miR172f expression in the *P. sativum* hairy roots of OE *LsBAHD3–13*. (**A**) Identifying overexpression of the *LsBAHD3* gene in the hairy roots of *P. sativum* via RT-PCR; (**B**) the expression level of miR172f in transgenic line OE *LsBAHD3–13* of *P. sativum* hairy roots. Asterisks denote significant differences at the level of *p* ≤ 0.01.

The transgenic *P. sativum* hairy roots line of OE *LsBAHD3–13* was confirmed via RT-PCR (Figure 1A), which demonstrated a single and discrete band with the correct size of about 1300 bp. RT-qPCR was performed to investigate the expression level of miR172f in OE *LsBAHD3–13 P. sativum* hairy roots, suggesting that the level was reduced significantly in OE *LsBAHD3-13* compared with the control (Figure 1B).

### 3.2. Confirmation That miR172f Targets the LsBAHD3 Gene

**Figure 2:**
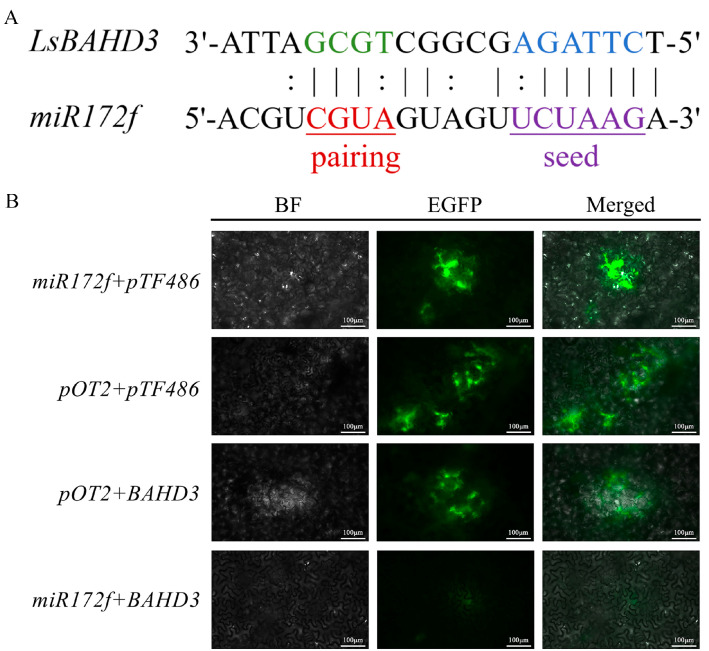
Identifying the interaction between miR172f and *LsBAHD3*. (**A**) predication of miR172f targets site in *LsBAHD3* gene; (**B**) verification of miR172f targeting in *LsBAHD3* gene via infiltration of *N. benthamiana* leaves with *A. tumefaciens* strain GV3101 carrying pTF486-BAHD3-eGFP and pOT2-miR172f. Scale bars represent 100 μm.

The psRNATarget prediction found that miR172f exhibits six consecutive complementary binding sites with the *LsBAHD3* gene, strongly suggesting that miR172f may target the *LsBAHD3* gene (Figure 2A). When *A. tumefaciens* strain GV3101 harboring the recombinant pTF486-BAHD3-eGFP and pOT2-miR172f was infiltrated in pairs into 5-week-old leaves of *N. benthamiana*, a significant fluorescence quenching effect was observed compared to the positive control (Figure 2B). These results convincingly suggested an interaction between miR172f and *LsBAHD3* in the transient *Nicotiana* leaf system, along with potential post-transcriptional regulation.

### 3.3. Knockdown miR172f Increases β-ODAP Content in the Hairy Roots of L. sativus

**Figure 3:**
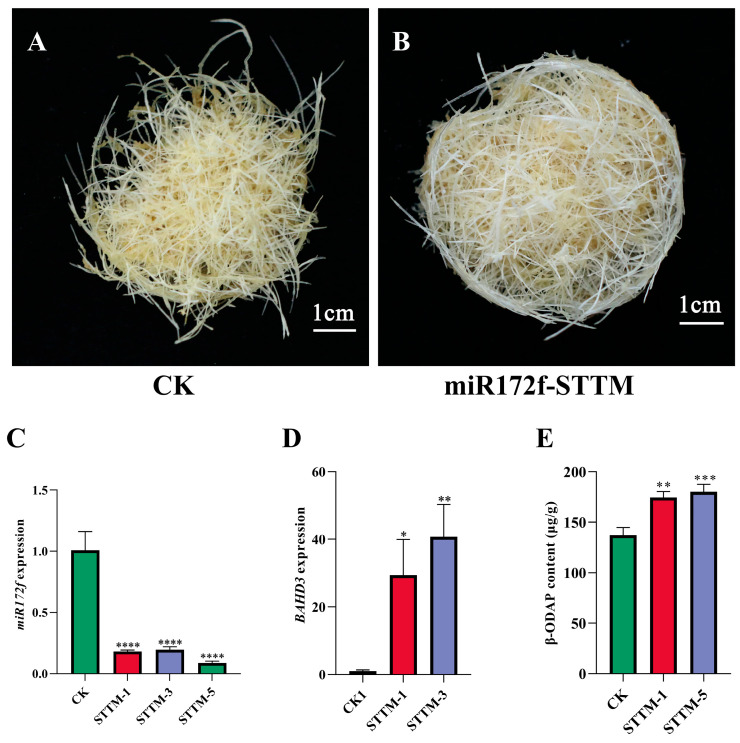
Determination of miR172f expression level and β-ODAP content in miR172f knocked-down hairy roots of *L. sativus*. (**A**) Hairy roots of *L. sativus* transformed with empty vector; (**B**) hairy roots of *L. sativus* with knocked-down miR172f; (**C**) identification of miR172f knocked-down hairy roots of *L. sativus* via RT-qPCR. (**D**) detection of the gene expression level of *LsBAHD3* via RT-qPCR in miR172f knocked-down hairy roots of *L. sativus*; (**E**) quantification of β-ODAP content in miR172f knocked-down lines. CK: hairy roots transformed with empty vector; STTM: miR172f knocked-down hairy roots. Asterisks denote significant differences at the level of 0.05 (*), 0.01 (**), 0.001 (***), or 0.0001 (****).

The hairy roots of *L. sativus* transformed with *pOT2-poly-cis* and *pFGC5941-pOT2-STTM-miR172f* were propagated separately in 1/2 MS liquid medium (Figure 3A,B). The expression level of knocked-down miR172f in the hairy roots was reduced significantly when compared with the control and analyzed via RT-qPCR (Figure 3C). In comparison with miR172f, the expression level of the *LsBAHD3* gene in miR172f-knockdown *L. sativus* hairy roots was significantly increased (Figure 3D), which suggested that the gene expression of *LsBAHD3* wa*s* upregulated by knocking down miR172f and further confirmed the targeting of miR172f to *LsBAHD3*. Then, the transgenic hairy roots of STTM-1 and STTM-5 were used to detect β-ODAP content. The results demonstrated greatly increased β-ODAP content in STTM-1 and STTM-5 when compared with the control (Figure 3E).

### 3.4. Transcriptomic Analysis of Knocked Down miR172f in the Hairy Roots of L. sativus

#### 3.4.1. GO and KEGG Analysis of DEGs Involved in β-ODAP Biosynthesis

To identify genes associated with β-ODAP biosynthesis in *L. sativus* hairy roots following miR172f knockdown, a transcriptomic analysis between STTM-1 and the control was conducted. PCA classified the STTM-1 and the control samples into two different groups, with the first two principal components (PC1 and PC2) accounting for 72.74% of the data variance (Figure S1), indicating that the PCA plot effectively captured most of the original information. Pearson’s correlation among the same groups was more than 0.95, suggesting a strong positive relationship in each group (Figure S2).

**Figure 4:**
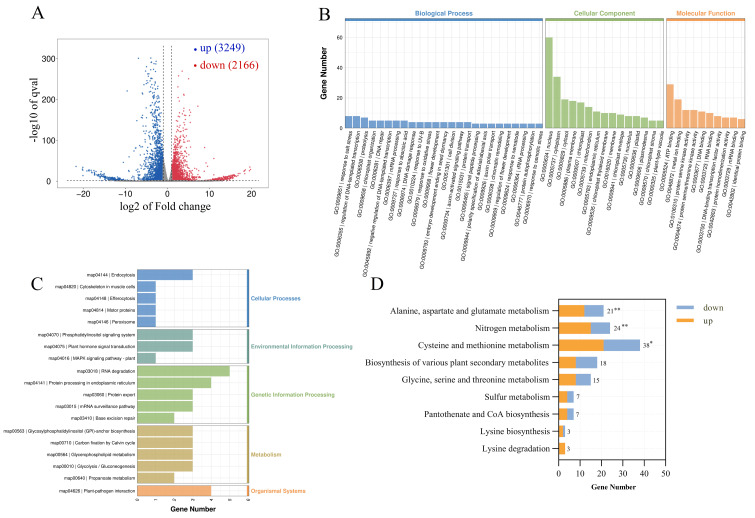
Transcriptomic assessments of knocked-down miR172f in the hairy roots of *L. sativus*. (**A**) Volcano plot of differentially expressed genes in miR172f knocked-down hairy roots of *L. sativus* compared to the control; (**B**) GO enrichment analysis of DEGs; (**C**) KEGG enrichment analysis of DEGs; (**D**) selected KEGG pathways potentially involved in β-ODAP biosynthesis. Asterisks denote significant differences at the level of 0.05 (*) or 0.01 (**).

A total of 5415 DEGs were identified, including 3249 up- and 2166 downregulated genes (Figure 4A). To determine the function of the DEGs, GO analysis was performed to classify the DEGs into biological processes (BP), cellular components (CC), and molecular functions (MF). The biological processes include defense response, regulation of DNA-templated transcription, and protein ubiquitination, and the cellular components include the nucleus, plasma membrane, cytoplasm, and chloroplast. The molecular functions are related to ATP binding, metal ion binding, DNA-binding transcription factor activity, DNA binding, protein serine kinase activity, protein serine/threonine kinase activity, and others (Figure 4B).

To determine the biochemical metabolic pathways associated with the DEGs, Kyoto Encyclopedia of Genes and Genomes enrichment (KEGG) analysis was performed. The DEGs were enriched mainly in plant hormone signal transduction, the MAPK signaling pathway-plant, phenylpropanoid biosynthesis, glycolysis/gluconeogenesis, starch and sucrose metabolism, cysteine and methionine metabolism (Figure 4C). Additionally, several pathways previously reported to be associated with β-ODAP biosynthesis were enriched in the KEGG analysis, including alanine, aspartate, and glutamate metabolism, nitrogen metabolism, cysteine and methionine metabolism, biosynthesis of various plant secondary metabolism, glycine, serine, and threonine metabolism, sulfur metabolism, pantothenate and CoA biosynthesis (Figure 4D).

#### 3.4.2. Function and Enrichment Analysis

**Figure 5:**
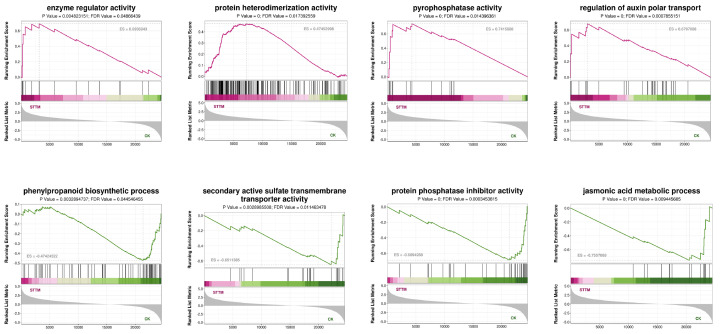
Gene set enrichment analysis (GSEA) of key pathways.

To further investigate the potential pathways involved in β-ODAP biosynthesis, GSEA was carried out based on GO terms. It was suggested that the increased β-ODAP content in miR172f knocked-down hairy roots of *L. sativus* was positively associated with the enriched upregulated gene sets including enzyme regulator activity, protein heterodimerization activity, pyrophosphatase activity, regulation of auxin polar transport and so on, while being negatively associated with the downregulated gene sets including phenylpropanoid biosynthetic process, secondary active sulfate transmembrane transporter activity, protein phosphatase inhibitor activity, jasmonic acid metabolic process, etc. (Figure 5).

#### 3.4.3. WGCNA and Module Identification

**Figure 6:**
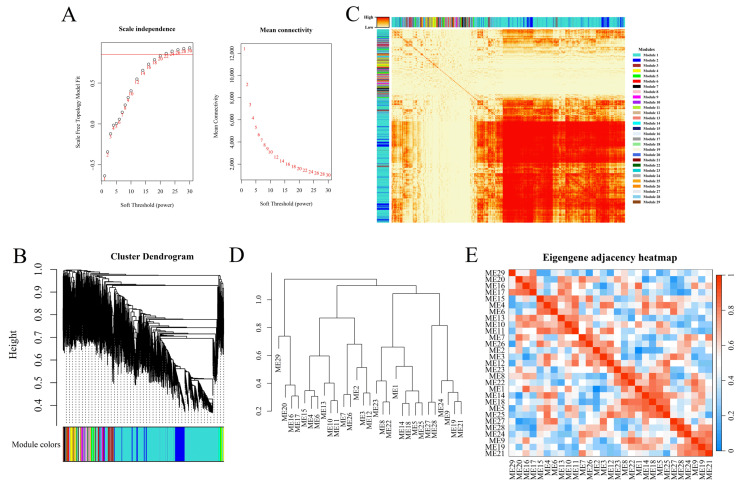
WGCNA based on the RNA-Seq data. (**A**) The soft threshold (β) was determined by scale independence and mean connectivity. The red line corresponds to 0.85. (**B**) Hierarchical cluster analysis indicates co-expression clusters with corresponding color assignments. Each color represents a module with the same gene expression pattern. (**C**) The correlation heatmap of the co-expression module genes. (**D**) Hierarchical clustering was performed on the feature genes of different modules. The smaller values on the y-axis reflect higher similarity between the two modules. (**E**) The heatmap of co-expression modules. Red represents a high correlation between the two modules, while blue represents significant differences in expression patterns.

The optimal soft threshold (β) was identified according to scale independence, and the mean connectivity was used to construct a reliable gene co-expression network, with no outlier sample excluded by cluster analysis (Figure 6A and Figure S3). While R^2^ was set to 0.85 and the soft threshold was set to 22, a total of 29 gene co-expression modules were identified (Figure 6B,C). Gene co-expression profiles were demonstrated in a heatmap, in which deeper colors represent a stronger connectivity between the two genes in the corresponding row and column (Figure 6C). Further hierarchical clustering, correlation heatmap analysis of different modules, and module–trait correlation analysis suggested that ME1 (module eigengene) was positively associated with the trait of knocked-down micro172f in hairy roots of *L. sativus* (correlation = 0.998164, *p* value = 0.000005), while ME2 was negatively associated with the trait (correlation = −0.97381, *p* value = 0.00102) (Figure 6D,E; Table S1). Moreover, ME1 showed significantly negative correlations with ME2 (correlation = −0.97, *p* value = 0.00095). Intriguingly, the *LsBAHD3* gene, which is the target gene of micro172f and a well-confirmed key gene in β-ODAP biosynthesis [3,15,19], was also found in ME1 with a high positive correlation with the trait of knocked-down micro172f in the hairy roots of *L. sativus* (correlation = 0.83, *p* value = 0.039, FDR = 0.069).

## 4. Discussion

The *Ls*BAHD3-*Ls*AAE3 module was reported to function conservatively in the β-ODAP biosynthesis of *L. sativus* and *P. sativum* [3,17]. When *Ls*BAHD3 was overexpressed in the hairy roots of *L. sativus* or *P. sativum*, β-ODAP content was significantly increased, suggesting that the activity of *Ls*BAHD3 catalyzes β-ODAP formation via oxalylation of L-DAP using oxalyl-CoA as the donor [3,15,19]. Intriguingly, the transgenic *P. sativum* hairy roots line of OE *LsBAHD3–13* reported by Zhang et al. [3] was identified via RT-PCR but failed via Western blot with tag antibody, which was thought to be regulated by post-translational modifications or microRNA-mediated gene silencing. MG132 treatment on the transgenic line of OE *LsBAHD3–13* caused significantly upregulated protein level of *Ls*BAHD*3*, suggesting the involvement of ubiquitin/26S proteasome system (UPS) in the regulation of β-ODAP biosynthesis [3].

The involvement of miRNAs in regulating the biosynthesis of secondary metabolites was well reported [21], which suggested the possible role of microRNAs in β-ODAP biosynthesis. The biosynthetic pathway of β-ODAP was strongly correlated with plant developmental stages and different environmental stress including drought and salinity [8], whereas miR172 family members play essential roles in these biological processes [27,28,29]. Combined with the downregulation of miR172f expression level and upregulation of β-ODAP content in miR172f knocked-down hairy roots of *L. sativus*, it could be concluded that miR172f was a novel regulator of β-ODAP level via targets of *LsBAHD3*.

The sulfur metabolism pathway serves as the fundamental biochemical scaffold for β-ODAP biosynthesis [11,12,13], and miR395 family members were involved in sulfur metabolism and its regulatory network via targets to ATPS (ATP sulfurylase), SLIM1 (Sulfur Limiting factor 1), and so on [24,25,26,31]. In this report, the transcriptomics response in miR172f-knockdown hairy roots of *L. sativus* was investigated. Several biological processes, including protein ubiquitination and metabolic pathways, including cysteine and methionine metabolism, were enriched via the GO/KEGG assay, which were highly consistent with the previous reports [3].

GSEA revealed that the enzyme regulator activity and protein heterodimerization activity were positively correlated with β-ODAP biosynthesis, whereas protein phosphatase inhibitor activity exhibited a negative correlation. Specifically, the enzymatic activity of serine O-acetyltransferase (SAT), which is the key enzyme involved in sulfur metabolism and β-ODAP biosynthesis, was typically regulated by its interaction with O-acetylserine(thiol)-lyase (OAS-TL) to form a hetero-oligomeric cysteine regulatory complex (CRC) [14,18,32]. Moreover, the calcium-dependent protein kinase *Ls*CDPK-SK5 was proven to phosphorylate *Ls*SAT2, which weakens the feedback inhibition of Cys on the activity of *Ls*SAT2 [33]. Moreover, the presence of *the LsBAHD3* gene in ME1, along with its strong positive correlation with the trait of micro172f knocked-down in the hairy roots of *L. sativus,* highlights the potentially functionally coherent gene set associated with β-ODAP biosynthesis.

## 5. Conclusions

**Figure 7:**
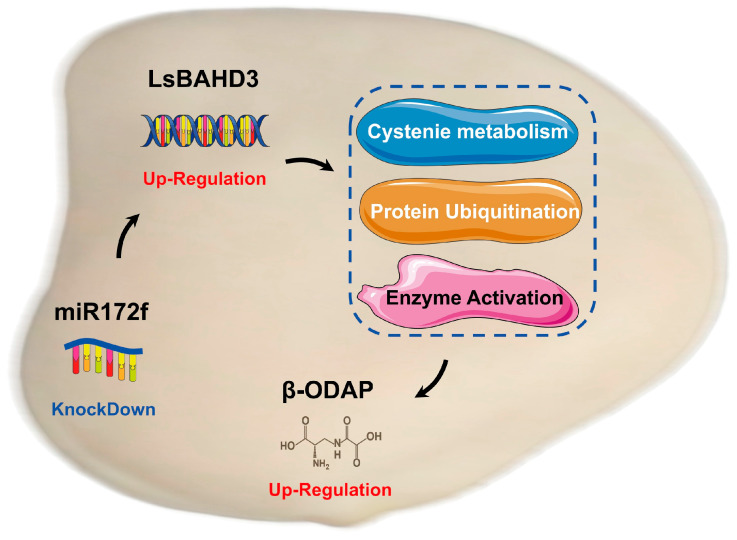
A proposed model illustrating that miR172f knocked-down in the hairy roots of *L. sativus* increased its β-ODAP content by affecting several biological processes and metabolic pathways.

In summary, miR172f was suggested as a novel regulator of β-ODAP biosynthesis based on the targeting of *LsBAHD3*, which encodes β-ODAP synthetase. The knocking down of miR172f in the hairy roots of *L. sativus* increased β-ODAP content by affecting several biological processes, including protein ubiquitination and metabolic pathways such as cysteine and methionine metabolism. Moreover, the enzyme regulator activity, protein heterodimerization activity, protein phosphatase inhibitor activity, and so on exhibited a strong correlation with β-ODAP biosynthesis (Figure 7). These results suggested that knocking down miR172f in the *L. sativus* hairy roots caused global re-programming (as 5415 genes). It would provide a novel target for the genetic manipulation of β-ODAP content in *L. sativus*, yet it needs to be further evaluated by social cell biology in situ methods. Ultimately, it provides a valuable insight into the genetic mechanisms related to β-ODAP biosynthesis, especially the gene expression regulation of β-ODAP synthetase in *L. sativus*.

## Figures and Tables

**Table 1:** Primers used in this study.

Name of Primer	Sequence of Primer (n 5′-3′ Orientation)
1300UBQ10-LsBAHD-F	CGACTCTAGAGGATCCATGCATCATCATCATCATCACAGTTCCATCCAAATCCTCTC
1300UBQ10-LsBAHD-R	CTAGTCTCGAGGTACCCTAACCAGAAGCAGCATCCATA
Stem-loop RT primer	GTCGTATCCAGTGCAGGGTCCGAGGTATTCGCACTGGATACGACTGCAGCAT
gma-miR172f-F	AGAATCTTGATGATGCTGCA
gma-miRNA-R	GTGCAGGGTCCGAGGT
U6-F	CATCCGATAAAATTGGAACGA
U6-R	TTTGTGCGTGTCATCCTTGCG

## Data Availability

All data supporting the findings of this study are available within the paper and within its Supplementary Materials published online.

## Supplementary Materials

The following supporting information can be downloaded at https://www.mdpi.com/article/10.3390/genes17030311/s1, Figure S1. The samples were classified into different groups based on PCA. Figure S2. High correlation between the two samples. Figure S3. Cluster analysis and sample outlier detection. Table S1. Module–trait correlation analysis.

## Abbreviations

The following abbreviations are used in this manuscript:

β-ODAP	β-N-oxalyl-L-α, β-diaminopropionic acid
SAT	Serine acetyltransferase
β-CAS	β-cyanoalanine synthase
BOS	β-ODAP synthetase
BAHD3	BAHD3 acyltransferase
STTM	Short tandem target mimics
GFP	Green fluorescent protein
DEG	Differentially expressed genes
PCA	Principal component analysis
WGCNA	Weighted gene co-expression network analysis
GSEA	Gene set enrichment analysis
