# Supplementary material for: Knocking Down miR172f in the Hairy Roots of Grass Pea Increases β-ODAP Content and Induces Global Transcriptomic Reprogramming
Source: Genes (Basel). 2026 Mar 9;17(3):311. doi: 10.3390/genes17030311 (PMC13025397; doi:10.3390/genes17030311)
Supplement: Supplementary file 1 [file genes-17-00311-s001.zip › supplementary files.pdf]

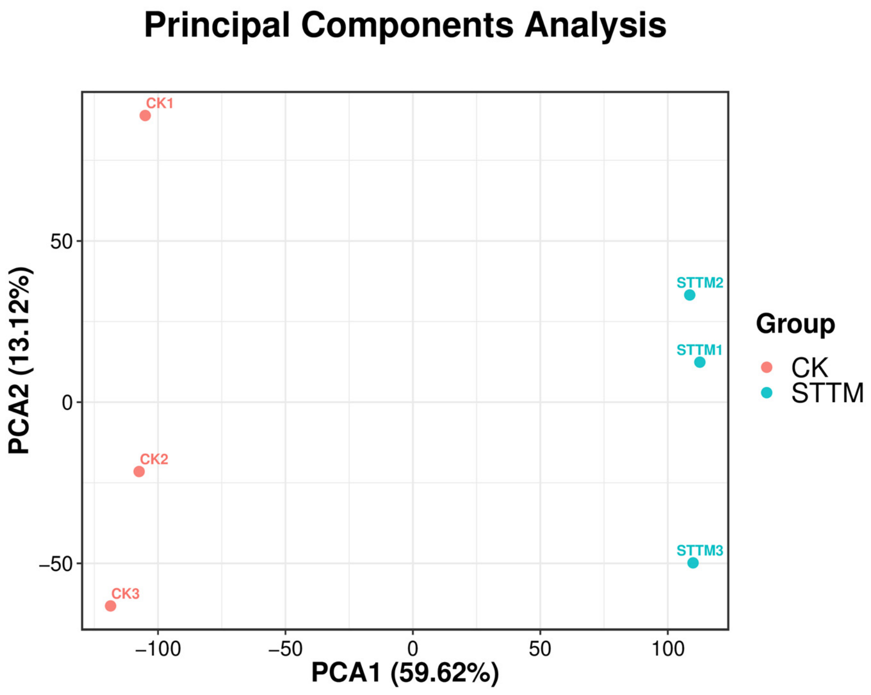

Figure S1. The samples classified into different groups based on PCA analysis.

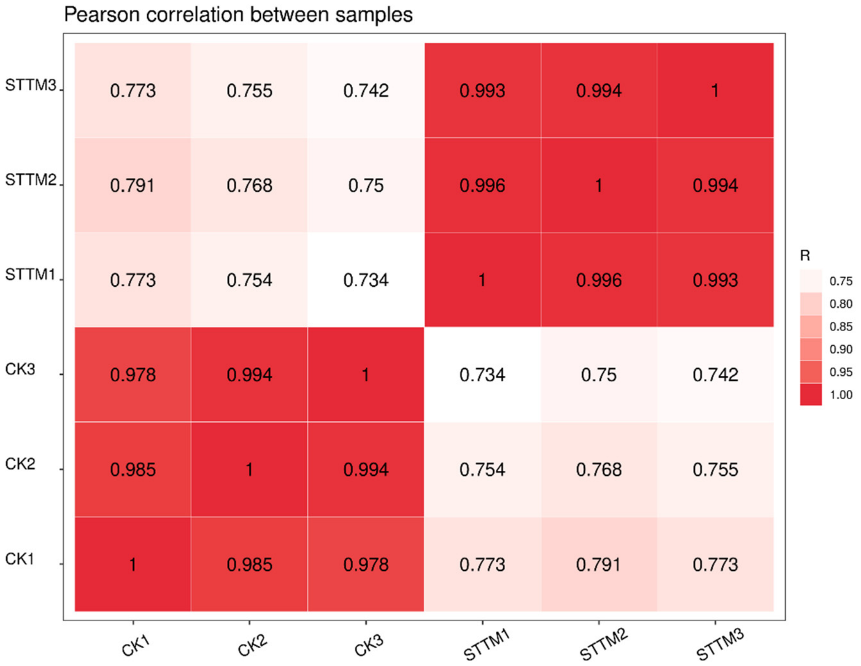

Figure S2. High correlation between each of the two samples.

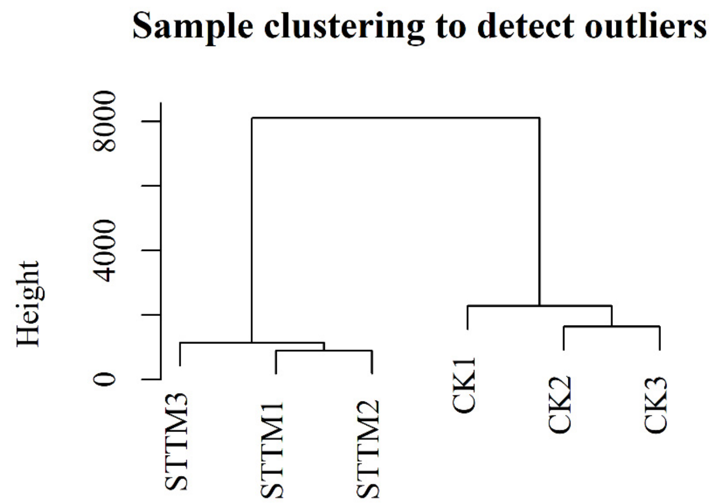

Figure S3. Cluster analysis and sample outlier detection.

Table S1. Module-trait correlation analysis

module	Group_cor	Group_pvalue
ME29	-0.00931	0.986035
ME20	-0.16146	0.759921
ME16	0.670188	0.145226
ME17	0.261668	0.616456
ME15	0.246647	0.637532
ME4	0.041945	0.937119
ME6	0.270542	0.604087
ME13	-0.05427	0.918672
ME10	0.4883	0.325764
ME11	-0.11587	0.82697
ME7	-0.28053	0.590248
ME26	-0.40691	0.423326
**ME2**	**-0.97381**	**0.00102**
ME3	-0.72911	0.100133
ME12	-0.43445	0.389323
ME23	-0.22576	0.667112
ME8	0.640333	0.170777
ME22	0.33998	0.509679
**ME1**	**0.998164**	**5.06E-06**
ME14	0.569308	0.238298
ME18	0.677866	0.138942
ME5	0.616998	0.191944
ME25	0.106981	0.84014
ME27	-0.26893	0.60633

ME28	-0.43125	0.39323
ME24	-0.27578	0.596817
ME9	0.593615	0.214166
ME19	0.445909	0.375467
ME21	0.075587	0.886836
